# Development and validation of a five-immune gene prognostic risk model in colon cancer

**DOI:** 10.1186/s12885-020-06799-0

**Published:** 2020-05-06

**Authors:** Haitao Chen, Jun Luo, Jianchun Guo

**Affiliations:** 1 Department of Orthopedic Surgery, Zhongnan Hospital of Wuhan University, Wuhan, 430071 China; 2 Department of Pathology, Zhongnan Hospital of Wuhan University, Wuhan, 430071 China; 3 Wuhan University Center for Pathology and Molecular Diagnostics, Wuhan, 430071 China

**Keywords:** Immune gene, Prognosis, Risk model, Colon cancer, TCGA

## Abstract

**Background:**

Colon cancer is a common and highly malignant cancer. Its morbidity is rapidly increasing, and its prognosis is poor. Currently, immunotherapy is a rapidly developing therapeutic modality of colon cancer. This study aimed to construct a prognostic risk model based on immune genes for the early diagnosis and accurate prognostic prediction of colon cancer.

**Methods:**

Transcriptomic data and clinical data were downloaded from The Cancer Genome Atlas database. Immune genes were obtained from the ImmPort database. Differentially expressed (DE) immune genes between 473 colon cancer and 41 adjacent normal tissues were identified. The entire cohort was randomly divided into the training and testing cohort. The training cohort was used to construct the prognostic model. The testing and entire cohorts were used to validate the model. The clinical utility of the model and its correlation with immune cell infiltration were analyzed.

**Results:**

A total of 333 DE immune genes (176 up-regulated and 157 down-regulated) were detected. We developed and validated a five-immune gene model of colon cancer, including LBP, TFR2, UCN, UTS2, and MC1R. This model was approved to be an independent prognostic variable, which was more accurate than age and the pathological stage for predicting overall survival at five years. Besides, as the risk score increased, the content of CD8+ T cells in colon cancer was decreased.

**Conclusions:**

We developed and validated a five-immune gene model of colon cancer, including LBP, TFR2, UCN, UTS2, and MC1R. This model could be used as an instrumental variable in the prognosis prediction of colon cancer.

## Background

Colon cancer is the third most common type of malignant tumor, which affects millions of people worldwide [1]. Despite significant advances that have been made for the treatment of colon cancer, its morbidity is rapidly increasing and its 5-year survival rate is low [2, 3]. Accordingly, to better the prognosis of colon cancer patients, it is essential and urgent to identify new indicators for the prognosis evaluation and targeted therapy of colon cancer.

The treatment of colon cancer has evolved to include not only the traditional methods of surgery, chemotherapy, and radiotherapy, but the rapidly developing immunotherapy [4]. It was also found that reduced immune cytotoxicity [5] and lack of T-cell infiltration [6] predict adverse outcomes in patients with colorectal carcinoma. Although immunotherapy has been reported to be effective in colon cancer with microsatellite instability [4], in contrast to other tumor types, inhibitors of PD-1/−L1 or CTLA 4 have not yet shown relevant efficacy in unselected colorectal cancer [7]. Also, because of the high heterogeneity of colon cancer [8], the prognosis may be considerably different between patients with similar clinical characteristics. Thus, it is essential to identify a multiple molecular model reflecting the sensitivity of patients to immunotherapy so that personalized treatments for colon cancer can be achieved.

In recent years, the development of high-throughput gene detection technology provides molecular markers for prognosis prediction and personalized treatment of colon cancer [9, 10]. However, as we know, none of these signatures were constructed based on multiple immune genes. Therefore, in the present study, we develop and validate a reliable prognostic model of colon cancer using differentially expressed (DE) immune genes, and verified the clinical utility of this model in colon cancer patients.

## Methods

### Database download

Transcriptomic data and clinical data were downloaded from The Cancer Genome Atlas (TCGA) database. Immune genes and Immune infiltrate data were downloaded from the ImmPort database (www.immport.org) and Tumor Immune Estimation Resource (TIMER) (http://cistrome.org/TIMER) [11], respectively.

### Identification of DE genes

The Wilcoxon signed-rank test was used to conduct differential analysis. Benjamini and Hochberg’s algorithm was applied to control the false discovery rate (FDR). Log_2_(fold change [FC]) > 1 and FDR < 0.05 were set as the cut-offs. Pheatmap package and gplots package was used to make heatmap and volcano map.

### Identification of DE immune genes

Based on the identified DE genes and immune gene list, the DE immune genes were detected using R software (v3.5.3). The pheatmap package and gplots package was used to make heatmap and volcano map.

### Function and pathway analysis of DE immune genes

The org.Hs.eg.db package and clusterProfiler package was used to conduct gene ontology (GO) analysis and Kyoto Encyclopedia of Genes and Genomes (KEGG) analysis. GO terms and KEGG terms were identified as significantly enriched when p.adjust < 0.05.

### Construction of the prognostic risk model

Based on DE immune genes in the training cohort, univariate analysis was performed to identify significant DE immune genes when *p* < 0.05. Then, Lasso regression was performed to eliminate genes that might overfit the model. Lastly, we applied multivariate analysis to identify the optimal prognostic immune genes for the model. The risk score was calculated based on a linear combination of the Cox coefficient and gene expression. The following calculation formula was used for the analysis:

$$ \mathrm{Risk}\ \mathrm{score}={\sum}_{\mathrm{i}=1}^{\mathrm{N}}\left(\mathrm{Expi}\ast \mathrm{Coei}\right) $$

N, Expi, and Coei represented gene number, level of gene expression, and coefficient value, respectively. The median was set as the cutoff value to divided all colon cancer patients into high-risk and low-risk groups. A high-risk score shows poor survival for colon cancer patients. Survival package and survminer package were used to conduct survival analysis. Time-dependent receiver operating characteristic (ROC) analysis for overall survival (OS) was used to evaluate the accuracy of the prognostic model. The survivalROC package was used to conduct a ROC analysis. An area under the ROC (AUC) > 0.60 was treated as an acceptable prediction value, and an AUC > 0.75 was considered as excellent for predictions [12, 13]. Risk score distribution plots, survival status scatter plots, and heatmap between the low-risk and high-risk groups were also applied to evaluate the model.

### Validation of the prognostic risk model

We used the testing cohort and the entire TCGA cohort to verify the accuracy of the prognostic risk model. Survival analysis and time-dependent ROC analysis were used to validate the model. Risk score distribution plots, survival status scatter plots, and heatmap was also used to evaluate the model.

### Independent prognostic value of the model in the entire cohort

To assess the prognostic value of the immune gene risk model, we applied both univariate and multivariate analyses of prognostic factors using Cox proportional hazards regression. Age, pathological stage, T, M, and N were treated as continuous variables. Gender was coded as female (0) or male (1). Factors in which *p* < 0.05 based on both univariate and multivariate analyses were identified as independent prognostic variables.

### Clinical utility of the model

To evaluate the prediction ability of the model in colon cancer patients, we assessed the relationships between our model (level of risk genes and the risk score) and the clinical features (age, gender, pathological stage, T, M, and N) in the entire cohort. Patients were separately divided into two groups according to age (> = 70 and < 70 years old), gender (female and male), stage (stage I&II and stage III&IV), T (T1–2 and T3–4), M (M0 and M1), and N (N0 and N1–3). Differences between the two groups were assessed with independent t-tests.

### Correlation between the model and immune cell infiltration

To understand whether the model could reflect the status of the tumor immune microenvironment in colon cancer patients, we evaluated the correlation between the risk score of the model and immune cell infiltration in the entire TCGA cohort. Pearson correlation coefficient test was used to estimate the relationship between the risk score of the model and the content of different types of immune cells.

## Results

### Basic information

**Fig. 1:**
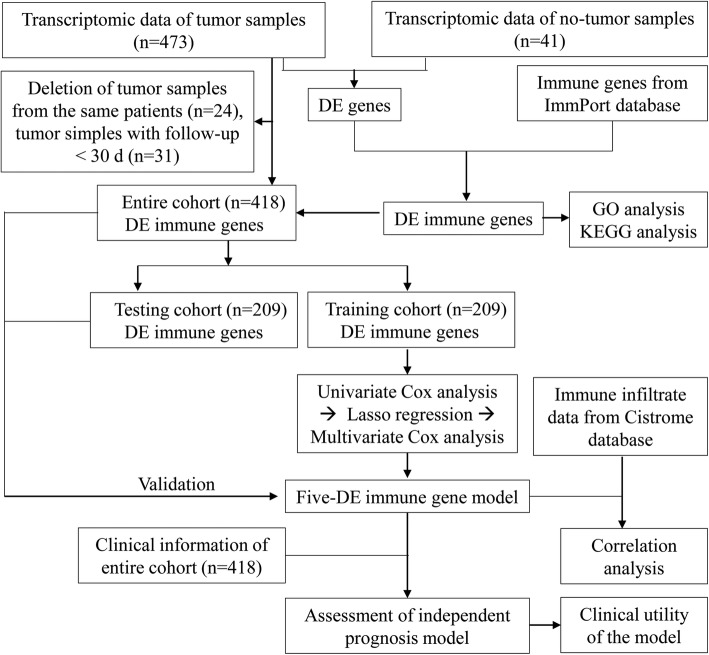
Diagram of the study. DE, differentially expressed; GO, gene ontology; KEGG, Kyoto Encyclopedia of Genes and Genomes; TIMER, Tumor IMmune Estimation Resource

Gene expression profile of 514 samples (473 tumors and 41 adjacent normals) for colon cancer patients was obtained from TCGA database. Twenty-four tumor samples coming from the same patients with other samples and thirty-one tumor samples with short follow-up time (< 30 days) were deleted. The clinical data of the remaining 418 tumor samples were shown in Table S1. Then these 418 tumor samples in the entire cohort were randomly classified into two groups, including a training cohort (*n* = 209) and a testing cohort (*n* = 209) (Table S2). The training cohort was applied to develop the prognosis risk model, and the testing cohort and the entire cohort were used to validate the model. The workflow of our study was illustrated in Fig. 1.

### Identification of DE genes

**Fig. 2:**
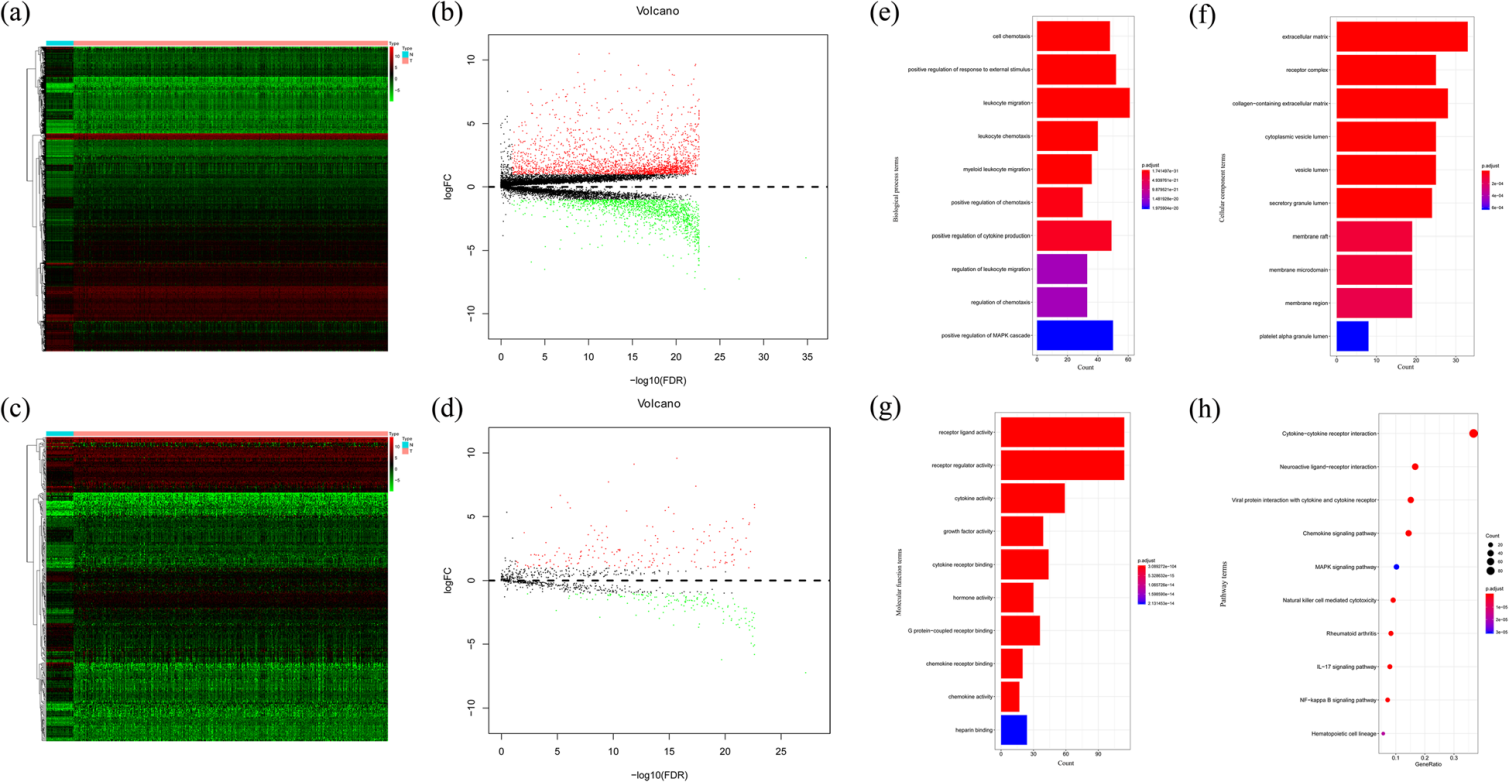
Identification of differentially expressed (DE) immune genes. **a** Heat map of the DE genes. **b** Volcano plot of the DE genes. **c** Heat map of the DE immune genes. **d** Volcano plot of the DE immune genes. **e** Biological process terms of the DE immune genes. **f** Cellular component terms of the DE immune genes. **g** Molecular function terms of the DE immune genes. **h** Kyoto Encyclopedia of Genes and Genomes (KEGG) analysis of the DE immune genes

3120 DE genes (1899 up-regulated and 1221 down-regulated) were recognized in colon cancer tissues compared with normal tissues. The DE genes were evaluated by the heatmap, as shown in Fig. 2a. The distribution of all DE genes according to the two dimensions of -log_10_FDR and log_2_FC was represented by a volcano map in Fig. 2b.

### Identification of the DE immune genes

A total of 1811 immune genes were downloaded from the ImmPort database. Based on DE genes and immune-related genes, 333 DE immune genes (176 up-regulated and 157 down-regulated) were detected. The top 10 up-regulated and down-regulated DE immune genes were shown in Table S3. The heatmap and volcano map of DE immune genes were depicted in Fig. 2c and d.

### Functional enrichment analysis of the DE immune genes

In total, 1775 GO terms, including 1690 biological process terms, 18 cellular component terms, and 67 molecular function terms were identified as significantly enriched. Likewise, 40 significantly enriched KEGG terms were detected. The top ten function and pathway terms were shown in Fig. 2e-h.

### Construction of a five-immune gene prognostic risk model

**Fig. 3:**
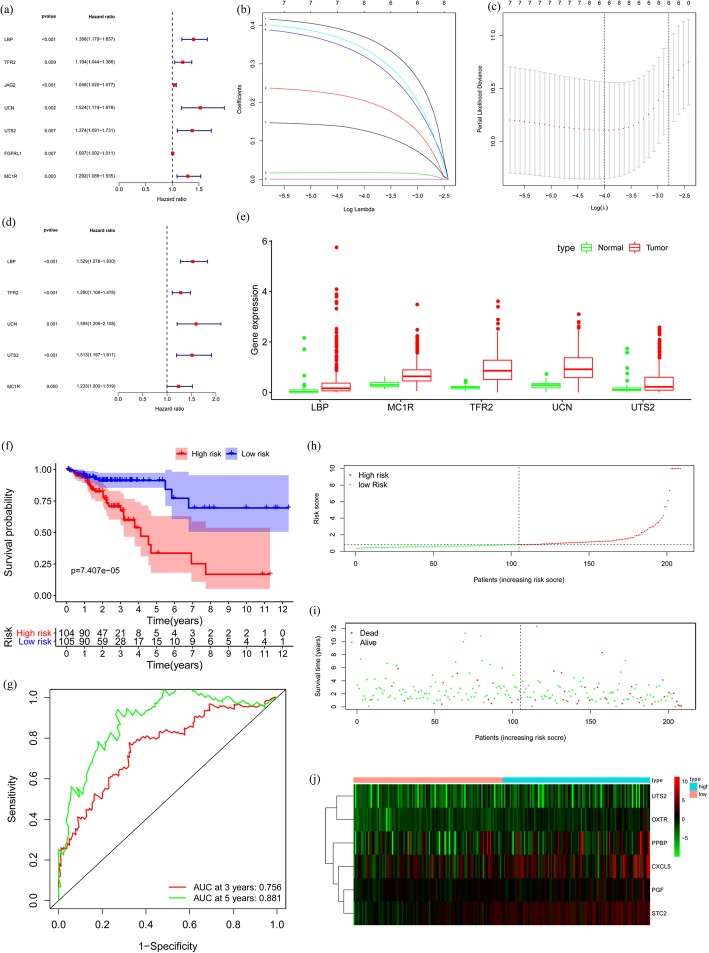
Construction of the prognostic risk model based on the training cohort. **a** Univariate Cox analysis. **b**, **c** Lasso regression. **d** Multivariate Cox analysis. **e** The expression value of the five DE immune genes in colon cancer patients. **f** Overall survival (OS). **g** Time-dependent receiver operating characteristic (ROC) curve analysis. **h** Risk score distribution. **i** Survival status scatter plots. **j** Heatmap of risk genes

Based on the training cohort, we screened seven immune genes that were possible prognostic genes using univariate Cox analysis (Fig. 3a). Then, we used Lasso regression to get six-candidate prognostic immune genes (Fig. 3b and c). Finally, we used multivariate Cox analysis to acquire five optimal immune genes, including lipopolysaccharide binding protein (LBP), transferrin receptor protein 2 (TFR2), urocortin (UCN), urotensin-II (UTS2), and melanocortin 1 receptor (MC1R) (Fig. 3d). All these five immune genes were high hazard genes, which were up-regulated DE genes (Fig. 3e). The formula for the risk score model was as follows: risk score = (0.4248 × expression value of LBP) + (0.2467 × expression value of TFR2) + (0.4666 × expression value of UCN) + (0.4139 × expression value of UTS2) + (0.209 × expression value of MC1R).

Based on the median risk score, all colon cancer patients were divided into a high-risk group (*n* = 104) and a low-risk group (*n* = 105). The time-dependent ROC analysis for OS was significantly different between the two risk groups (*p* = 7.407e-05) (Fig. 3f). The median survival time of the low-risk group was more than ten years, while that of the high-risk group was less than five years. Also, the 3- and 5-year survival rates of the low-risk group were 91 and 91%, respectively, whereas the corresponding rates in the high-risk group were 72 and 35%. The AUC values for the five-immune gene prognostic model at three and five years of OS was 0.756 and 0.881, respectively (Fig. 3g). The risk score distribution plot, survival status plot, and the heatmap between two groups was shown in Fig. 3h-j.

### Validation of the risk model

**Fig. 4:**
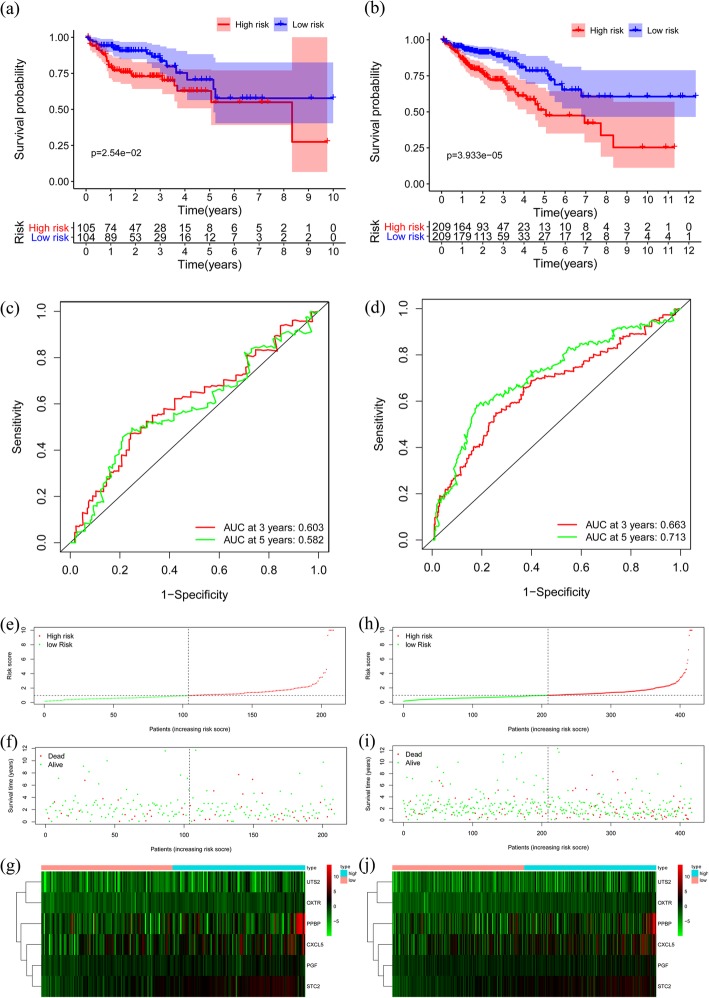
Validation of the prognostic model in the testing and entire cohort. **a** Overall survival (OS) in the testing cohort. **b** OS in the entire cohort. **c** Time-dependent receiver operating characteristic (ROC) curve analysis in the testing cohort. **d** Time-dependent ROC curve analysis in the entire cohort. **e** Risk score distribution in the testing cohort. **f** Survival status scatter plots in the testing cohort. **g** Heatmap of risk genes in the testing cohort. **h** Risk score distribution in the entire cohort. **i** Survival status scatter plots in the entire cohort. **j** Heatmap of risk genes in the entire cohort

To validate the accuracy of the risk model, we analyzed the model in the testing cohort and the entire TCGA cohort. The risk score of each patient in the testing and the entire cohort was calculated and then divided into two groups based on the median. In the testing cohort, 104 patients and 105 patients were categorized as high-risk and low-risk groups, respectively. Similarly, in the entire cohort, 209 patients and 209 patients were divided into high-risk and low-risk groups, respectively. There were significant differences in survival curves between the two risk groups in the testing cohort and the entire cohort (*p* < 0.05) (Fig. 4a-b). The AUC values at 3- and 5-year were 0.603 and 0.582 in the testing cohort, respectively (Fig. 4c). The AUC values at 3- and 5-year were 0.663 and 0.713 in the entire cohort, respectively (Fig. 4d). The risk score distribution plot, survival status plot, and heatmap of risk gene expression in the two cohorts were presented in Fig. 4e-j. All risk gene level was higher in the high-risk group than that in the low-risk group, which showed that the risk model could accurately predict the prognosis of colon cancer patients.

### Independent prognostic value of the risk model

**Fig. 5:**
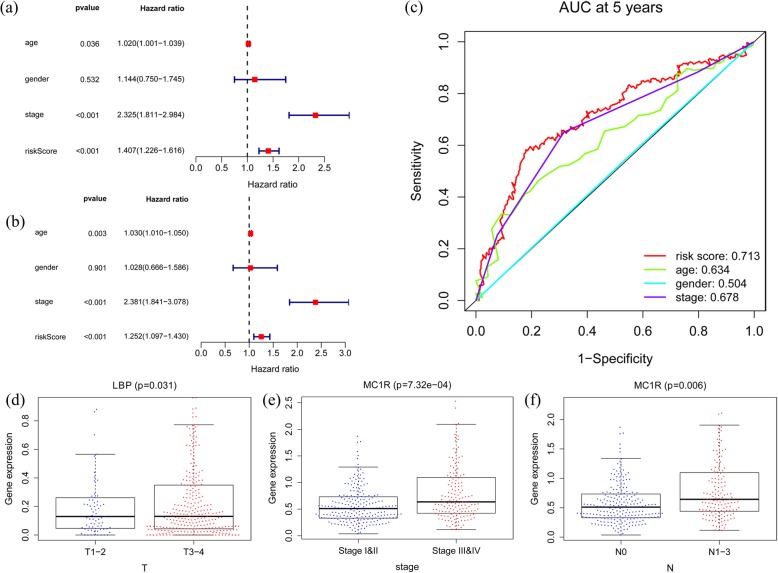
Independent prognostic value of the model in the entire cohort. **a** Univariate analysis. **b** Multivariate Cox analyses. **c** Time-dependent receiver operating characteristic (ROC) curve analyses of the prognostic variables in the entire cohort at five years. **d** Relationship between LBP and T category. **e** Relationship between MC1R expression and pathological stage. **f** Relationship between MC1R and N category

Both the univariate analysis and the multivariate analysis revealed that age, the pathological stage, and the risk score were related to OS in the entire cohort (*p* < 0.05) (Fig. 5a-b). These results indicated that age, the pathological stage, and the prognostic risk model could be used independently to predict the prognosis of colon cancer patients. We then further compared these variables and found that the risk score was more accurate than the pathological stage and age in predicting OS at five years. The AUCs at five years for the risk score, age, and the pathological stage were 0.713, 0.634 and 0.678, respectively (Fig. 5c).

### Clinical utility of the model

When the values of LBP increased, the T category of colon cancer patients increased in the entire cohort (*p* < 0.05) (Fig. 5d). Similarly, as the values of MC1R increased, the value of the pathological stage and N category increased (*p* < 0.05) (Fig. 5e-f). These results proved that the immune gene expression of the model is related to the development of colon cancer.

### Correlation between the risk model and immune cell infiltration

**Fig. 6:**
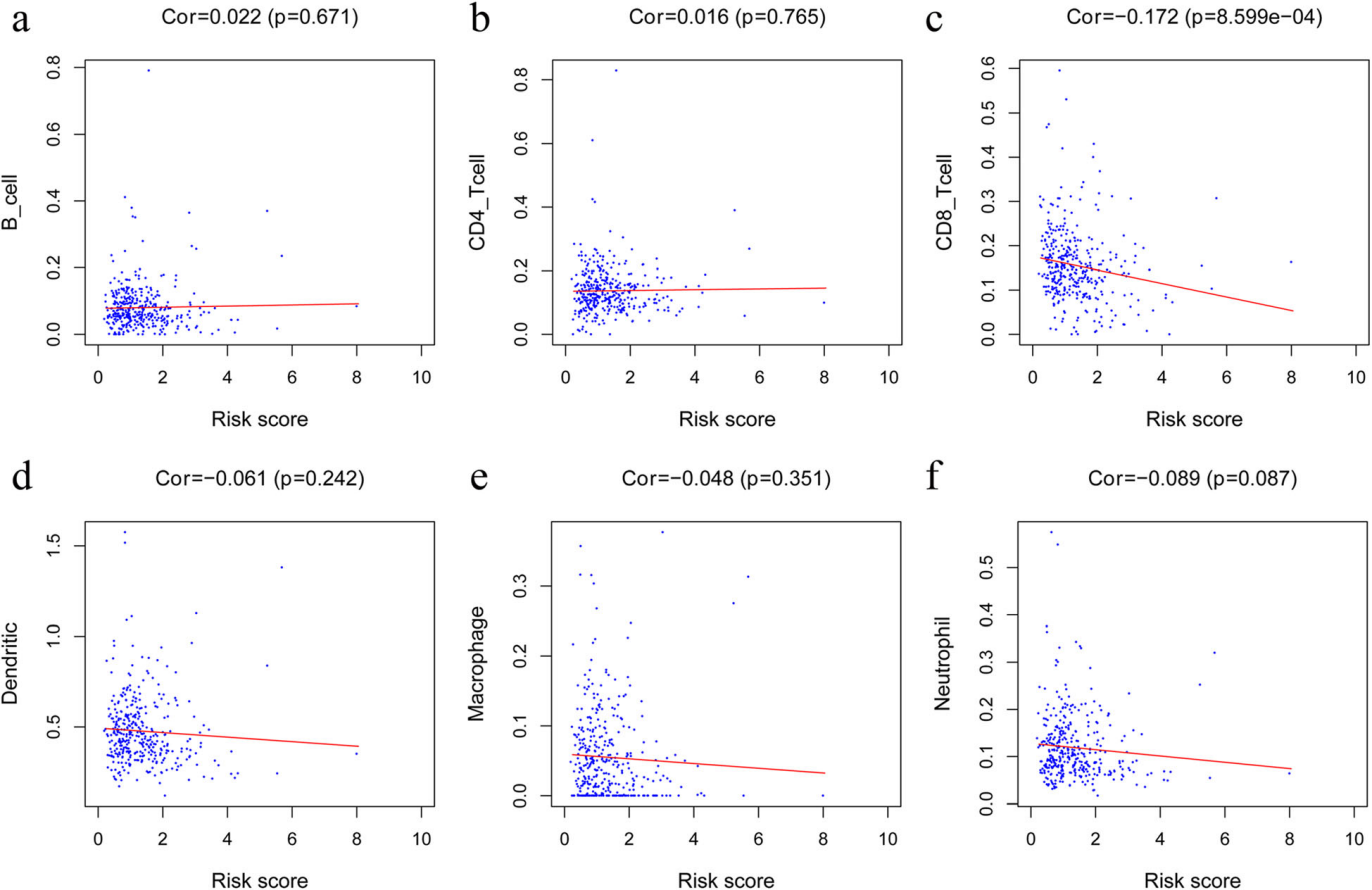
Correlation analysis between the risk score and immune cell infiltration. **a** B cells. **b** CD4^+^ T cells. **c** CD8^+^ T cells. **d** Dendritic cells. **e** Macrophages. **f** Neutrophils

The risk score was negatively correlated with the content of CD8^+^ T cells in colon cancer tissues in the entire cohort (*p* < 0.05) (Fig. 6). The result demonstrated that the immune gene model might reflect the status of the tumor immune microenvironment in colon cancer patients.

## Discussion

Colon cancer is one of the most common carcinomas worldwide, responsible for about 1,100,000 new cases and 550,000 deaths in 2018 [14]. Several studies have reported the role of the immune gene in the initiation and progression of carcinoma [15, 16]. In the current study, we established and validated a prognostic model based on five DE immune genes, which could be used as an independent prognostic variable. We found that this model could provide more accurate predictive value than the pathological stage and age in predicting OS at five years. Additionally, the immune gene model could reflect the tumor immune microenvironment according to the correlation analysis between the model and immune cell infiltration. Besides, we conducted an enrichment analysis of function and pathway of the DE immune genes, which might provide a reference for further basic research in colon cancer.

In the current study, we developed a prognostic risk model based on five DE immune genes, named LBP, TFR2, UCN, UTS2, and MC1R. Firstly, this model was constructed by five DE immune genes between colon cancer and normal tissues. These DE immune genes might reflect the progression of colon cancer, which could contribute to the early diagnosis of colon cancer. Secondly, multiple algorithms were applied for model selection, and the prediction value of the model had also been confirmed, which proved the accuracy and dependability of the prognostic model. Besides, these DE immune genes may have great promise to be novel molecular targets in immunotherapy. LBP, as a pattern recognition protein, can activate the cell to produce cytokines when faced with various microbial ligands [17]. Serum LBP was also proved to be a useful prognostic parameter for breast cancer patients after radiation therapy [18]. TFR2, which play a crucial role in the regulation of iron homeostasis, was found high expression in human colon cancer cell [19, 20]. UCNs are corticotropin-releasing factor-related peptides, regulating gastrointestinal motor and visceral pain during stress [21]. UTS2 was recently used as a new drug target towards colon cancer cells [22]. Individuals carrying MC1R variants are associated with a higher risk of melanoma, and MC1R had been used as an intervention target for melanoma [23].

To assess the prediction capability of the model, we analyzed several clinical variables as well as the risk score. Age, the pathological stage, and the risk score were identified as independent prognostic variables. Age is a prominent risk factor for multiple tumors including colorectal cancer [24], which was in line with the results predicted by the model. Further comparison showed that the predicted value of the model is better than age and the pathological stage. Thus, our model showed a high prediction ability. To evaluate the clinical applicability of the model, we analyzed the relationships between factors in the model and certain clinical variables. We found that higher gene expression of the immune genes in the model was highly correlated with higher pathological stage, which, on the other hand, verifies the reliability of our prognostic model. The previous study has reported that immune infiltration is vital in response to treatment and prognosis of colon cancer [25]. Galon et al. [6] reported that individual immune cell markers have prognostic impacts on patients who have colon cancer. It was reported that the inhibition of CD8^+^ T cells was associated with enhanced tumor progression, and mesenchymal stromal cells PD-L1 could promote colon cancer by inhibiting the antitumor immune responses of CD8^+^ T cell [26]. In the present study, we discovered that the risk score was negatively related to the infiltration of CD8+ T cells. These results might also confirm that our model was reliable in predicting the prognosis of colon cancer.

In the present study, we conducted an enrichment analysis of the DE immune genes. Leukocyte migration, cell chemotaxis, extracellular matrix, and receptor regulatory activity were enriched GO terms. Solid tumor sample from TCGA comprises both tumor and other cells, among which immune cell play vital roles in the development of the tumor. Chu et al. [27] reported that leukocyte migration is a natural process, from blood to tissue through the vascular barrier, to deal with the invasion of pathogens, which might reflect the status of tumor microenvironment. The distinct biochemical and biophysical properties of extracellular matrix can influence cell phenotype, and the dysregulation of extracellular matrix dynamics leads to the development of cancer [28]. KEGG analysis found that cytokine-cytokine receptor interaction, chemokine signaling pathway, and MAPK signaling pathway were significant pathways. Cytokine-cytokine receptor interaction was related to the viability of colon cancer cell lines [29]. Besides, previous studies have demonstrated that abnormally activated MAPK pathway is closely associated with growth and metastasis of colon cancer [30, 31]. These significantly enriched functions and pathways may provide a reference for further basic experiments.

The current study has several advantages. Firstly, we constructed a prognostic model of colon cancer based on DE immune genes for the first time. Secondly, we created the model using various statistical methods and validated the model using the testing cohort and the entire cohort. Thus, the prognostic risk model for colon cancer patients was accurate and reliable. Thirdly, the risk score model could be used as an independent prognostic index, which was more accurate than the pathological stage and age in predicting OS. Finally, our model could also be used to predict immune cell infiltration in the progression of colon cancer.

The present study has limitations. Firstly, we developed the prognostic risk model based on public databases, which was not verified by prospective clinical trials. Additionally, the underlying mechanisms of how the detected DE immune genes impact the progress of colon cancer require further study by basic experiments.

## Conclusion

We developed and validated a five-immune gene model of colon cancer, including LBP, TFR2, UCN, UTS2, and MC1R. This model could be used as an instrumental variable in the prognosis prediction of colon cancer.

## Supplementary information

**Additional file 1: Table S1.** Clinical information of the 418 colon cancer patients in the entire cohort.

**Additional file 2: Table S2.** Grouping of the colon cancer patients.

**Additional file 3: Table S3.** The top 10 up-regulated and down-regulated DE immune genes.

## Data Availability

All analyzed data are included in this published article and its supplementary information file. The original data are available upon reasonable request to the corresponding author.

## Abbreviations

DE: Differentially expressed
TCGA: The Cancer Genome Atlas
FDR: False discovery rate
FC: Fold change
GO: Gene ontology
KEGG: Kyoto Encyclopedia of Genes and Genomes
ROC: Receiver operating characteristic
OS: Overall survival
AUC: Area under the curve
