# Supplementary material for: Development and validation of a five-immune gene prognostic risk model in colon cancer
Source: BMC Cancer. 2020 May 6;20:395. doi: 10.1186/s12885-020-06799-0 (PMC7204296; doi:10.1186/s12885-020-06799-0)
Supplement: Supplementary file 1 — Additional file 1: Table S1. Clinical information of the 418 colon cancer patients in the entire cohort. [file 12885_2020_6799_MOESM1_ESM.docx]

**Table S1** Clinical information of the 418 colon cancer patients in the entire cohort

| Clinical Traits | Variable | N (Total=418) | Percentage (%) |
| --- | --- | --- | --- |
| Survival status | Alive | 330 | 78.9 |
|  | Dead | 88 | 21.1 |
| Age (years) | <70 | 229 | 54.8 |
|  | >=70 | 189 | 45.2 |
| Gender | Female | 193 | 46.2 |
|  | Male | 225 | 53.8 |
| Pathological stage | Stage I | 72 | 17.3 |
|  | Stage II | 166 | 39.7 |
|  | Stage III | 123 | 29.4 |
|  | Stage IV | 57 | 13.6 |
| T | T1 | 9 | 2.2 |
|  | T2 | 74 | 17.7 |
|  | T3 | 285 | 68.2 |
|  | T4 | 49 | 11.7 |
|  | TX | 1 | 0.2 |
| M | M0 | 315 | 75.4 |
|  | M1 | 58 | 13.9 |
|  | TX | 45 | 10.7 |
| N | T0 | 248 | 59.3 |
|  | T1 | 98 | 23.4 |
|  | T2 | 72 | 17.3 |
