# Supplementary material for: Development and validation of a five-immune gene prognostic risk model in colon cancer
Source: BMC Cancer. 2020 May 6;20:395. doi: 10.1186/s12885-020-06799-0 (PMC7204296; doi:10.1186/s12885-020-06799-0)
Supplement: Supplementary file 2 — Additional file 2: Table S2. Grouping of the colon cancer patients. [file 12885_2020_6799_MOESM2_ESM.docx]

**Table S2** Grouping of the colon cancer patients

| Clinical Traits | Variable | Training Cohort | Testing Cohort | Entire Cohort |
| --- | --- | --- | --- | --- |
| Survival status | Alive | 168 (40.1%) | 162 (38.8%) | 330 (78.9%) |
|  | Dead | 41 (9.8%) | 47 (11.3%) | 88(21.1%) |
