# Supplementary material for: Development and validation of a five-immune gene prognostic risk model in colon cancer
Source: BMC Cancer. 2020 May 6;20:395. doi: 10.1186/s12885-020-06799-0 (PMC7204296; doi:10.1186/s12885-020-06799-0)
Supplement: Supplementary file 3 — Additional file 3: Table S3. The top 10 up-regulated and down-regulated DE immune genes. [file 12885_2020_6799_MOESM3_ESM.docx]

**Table S3** The top 10 up-regulated and down-regulated DE immune genes

| Type | Genes | LogFC | P Value | FDR |
| --- | --- | --- | --- | --- |
| Up-regulated | SLC10A2 | -7.24754 | 8.62E-32 | 5.87E-28 |
|  | INSL5 | -6.22224 | 1.23E-21 | 1.92E-20 |
|  | PYY | -5.81648 | 5.41E-26 | 2.24E-23 |
|  | GUCA2A | -5.1536 | 1.08E-25 | 2.70E-23 |
|  | BMP3 | -5.06109 | 3.52E-26 | 2.22E-23 |
|  | SST | -4.93055 | 2.96E-25 | 4.39E-23 |
|  | CHGA | -4.38012 | 1.52E-25 | 3.19E-23 |
|  | CHP2 | -3.88542 | 5.71E-25 | 6.13E-23 |
|  | GLP2R | -3.73714 | 2.56E-26 | 2.22E-23 |
|  | CNTFR | -3.69115 | 1.29E-23 | 4.91E-22 |
| Down-regulated | INHBA | 5.702328 | 4.89E-26 | 2.22E-23 |
|  | ESM1 | 5.955085 | 2.66E-26 | 2.22E-23 |
|  | ZC3HAV1L | 2.273621 | 3.74E-25 | 5.05E-23 |
|  | ULBP2 | 4.498442 | 6.41E-25 | 6.33E-23 |
|  | S100A2 | 4.372702 | 7.51E-25 | 6.93E-23 |
|  | TRIM27 | 1.003345 | 8.14E-25 | 7.15E-23 |
|  | APLN | 3.303721 | 1.74E-24 | 1.24E-22 |
|  | MET | 2.056308 | 1.76E-24 | 1.25E-22 |
|  | UCN2 | 4.835996 | 4.22E-24 | 2.35E-22 |
|  | FGFRL1 | 2.437212 | 8.36E-24 | 3.76E-22 |

Abbreviations: DE differentially expressed, FC Fold Change, FDR false discovery rate
